# The impact of weather changes on air quality and health in the United States in 1994–2012

**DOI:** 10.1088/1748-9326/10/8/084009

**Published:** 2015-08-12

**Authors:** Iny Jhun, Brent A Coull, Joel Schwartz, Bryan Hubbell, Petros Koutrakis

**Affiliations:** 1 Department of Environmental Health, Harvard School of Public Health, 401 Park Drive, Boston, MA 02215, USA; 2 Department of Biostatistics, Harvard School of Public Health, 655 Huntington Avenue, Boston, MA 02115, USA; 3 Office of Air Quality, Planning, and Standards, US Environmental Protection Agency, 109 TW Alexander Drive, Research Triangle Park, NC27711, USA

**Keywords:** air pollution, weather, trend analysis, ozone, fine particulate matter, mortality

## Abstract

Air quality is heavily influenced by weather conditions. In this study, we assessed the impact of long-term weather changes on air quality and health in the US during 1994–2012. We quantified past weather-related increases, or ‘weather penalty’, in ozone (O_3_) and fine particulate matter (PM_2.5_), and thereafter estimated the associated excess deaths. Using statistical regression methods, we derived the weather penalty as the additional increases in air pollution relative to trends assuming constant weather conditions (i.e., weather-adjusted trends). During our study period, temperature increased and wind speed decreased in most US regions. Nationally, weather-related 8 h max O_3_ increases were 0.18 ppb per year (95% CI: 0.06, 0.31) in the warm season (May–October) and 0.07 ppb per year (95% CI: 0.02, 0.13) in the cold season (November–April). The weather penalties on O_3_ were relatively larger than PM_2.5_ weather penalties, which were 0.056 µg m^−3^ per year (95% CI: 0.016, 0.096) in warm months and 0.027 µg m^−3^ per year (95% CI: 0.010, 0.043) in cold months. Weather penalties on O_3_ and PM_2.5_ were associated with 290 (95% CI: 80, 510) and 770 (95% CI: 190, 1350) excess annual deaths, respectively. Over a 19-year period, this amounts to 20 300 excess deaths (5600 from O_3_, 14 700 from PM_2.5_) attributable to the weather penalty on air quality

## 1 Introduction

Air quality is significantly influenced by both emissions and weather conditions. Among pollutants, ozone (O_3_) and fine particulate matter (PM_2.5_) have been extensively studied, as O_3_ and PM_2.5_ exposures are associated with a wide range of adverse health outcomes, including respiratory illnesses, hospital admissions, and premature mortality (Jerrett *et al* 2009, Krewski *et al* 2009, US EPA 2010). Studies evaluating the efficacy of O_3_ and PM_2.5_ mitigation efforts have utilized statistical methods to account for the effects of weather-related variations and simultaneously derived the relationships between air pollution and weather parameters (Cox and Chu 1993, Bloomfield *et al* 1996, Cox and Chu 1996, Thompson *et al* 2001, Camalier *et al* 2007, Zheng *et al* 2007). In this study, we built upon prior methods to directly quantify the impact of long-term weather conditions on air pollution changes in the US.

The influence of weather conditions on O_3_ and PM_2.5_ differ substantially, which affects both the magnitude and uncertainty of the impact of long-term weather changes on these two pollutants. The effect of ground-level weather conditions on O_3_ is generally more robust and better characterized than that on PM_2.5_. O_3_ primarily results from reactions between nitrogen oxides and volatile organic compounds in the presence of sunlight, and high temperature, low humidity, and low wind speed conditions favor O_3_ formation (The National Academies Press 1991). Weather impacts on PM_2.5_ can be more variable, given the diversity of particle components (e.g., sulfate, nitrate, organic carbon, and elemental carbon). In general, particles are efficiently scavenged through wet deposition (Balkanski *et al* 1993), but other weather impacts can be more complex. For instance, rising temperatures can increase oxidation and production of sulfate particles (Dawson *et al* 2007b), but reduce nitrate particles through volatilization from particle to gas phase (Seinfeld and Pandis 2006).

**Figure 1:**
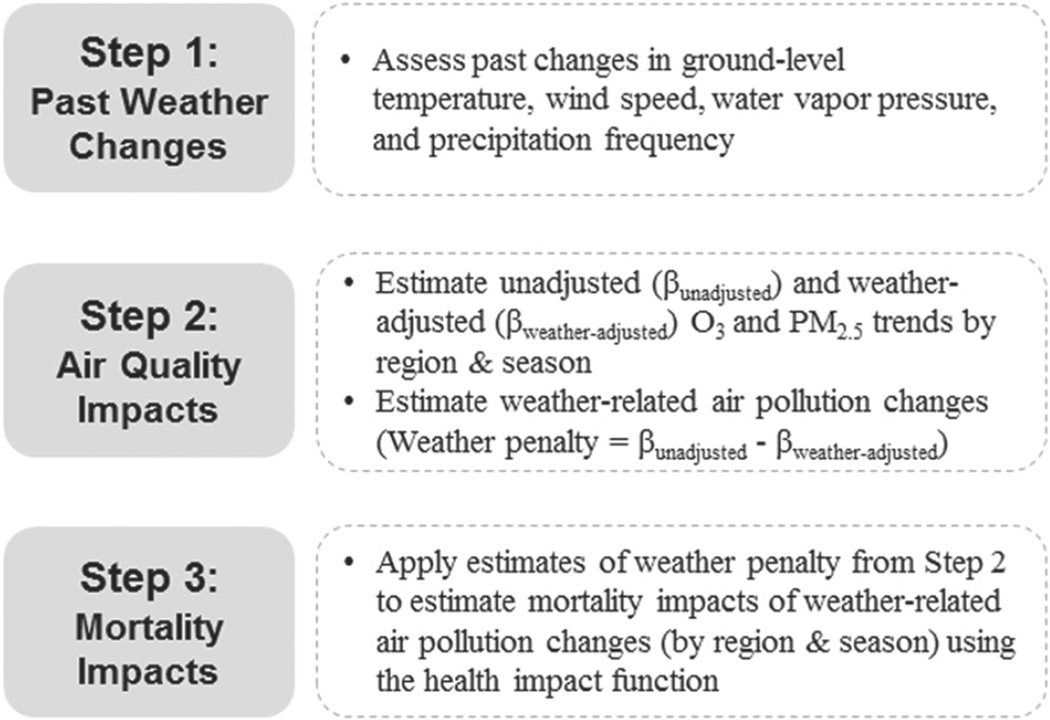
Schematic illustration of stages of analyses.

In this study, we employed statistical regression methods to quantify the effect of ground-level weather changes, or ‘weather penalty’, on recent O_3_ and PM_2.5_ trends in the US (1994–2012). We then applied mortality risk estimates from epidemiological studies to estimate the excess mortalities associated with the weather penalty on O_3_ and PM_2.5_ (see figure 1 for schematic illustration). Specifically, we analyzed the impact of changes in temperature, wind speed, water vapor pressure, and precipitation frequency, as prior studies have identified these parameters to be among the most important meteorological determinants of O_3_ and PM_2.5_ concentrations (Thompson *et al* 2001, Dawson *et al* 2007a, 2007b, Tai *et al* 2010).

## 2. Materials and methods

### 2.1. Air quality data collection

**Figure 2:**
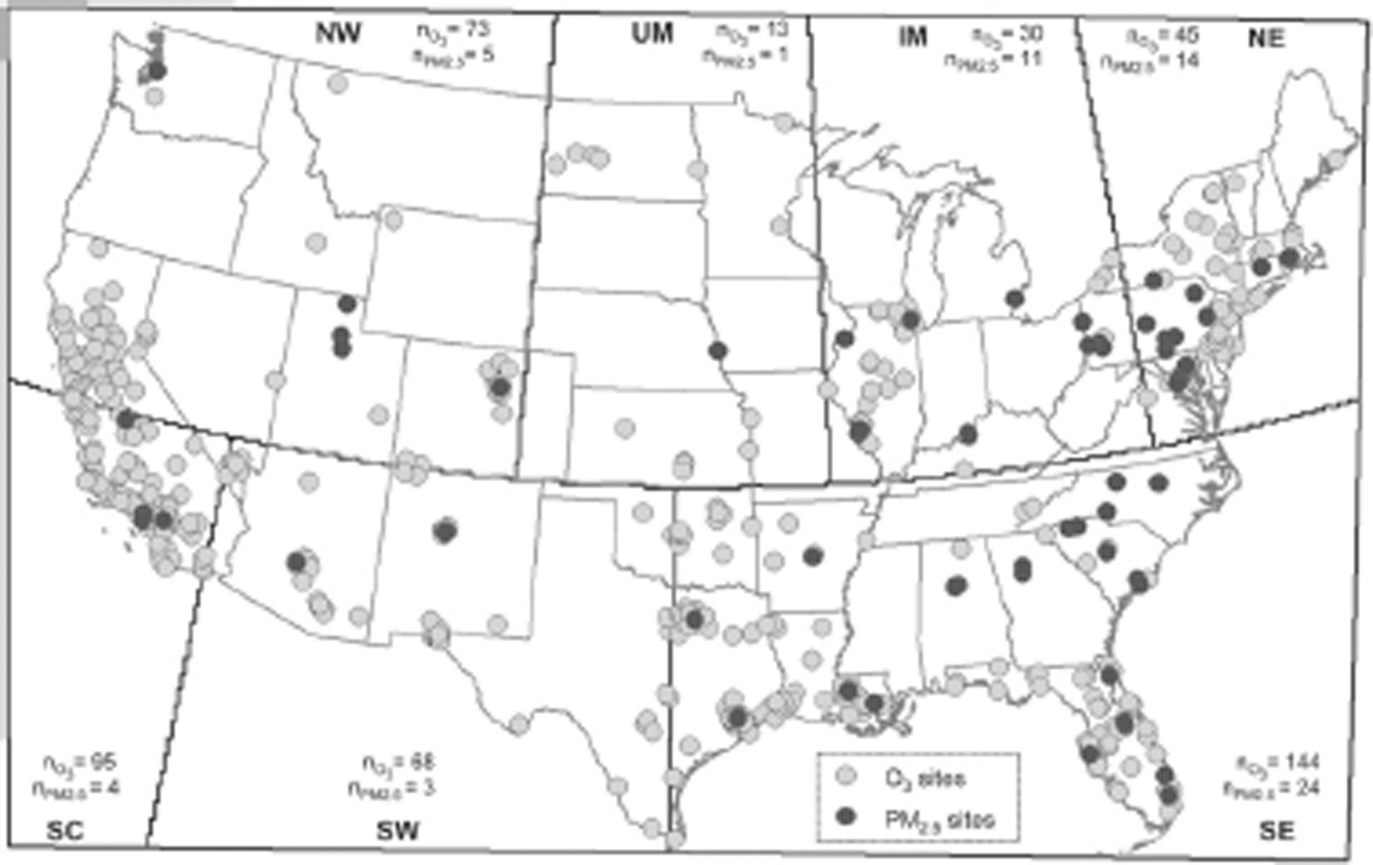
Pollutant monitoring site locations. Number of O_3_ and PM_2.5_ monitoring sites are noted by *n*_O3_ and *n*_PM2.5_, respectively (NW: Northwest, UM: Upper Midwest, IM: Industrial Midwest, NE: Northeast, SC: Southern California, SW: Southwest, SE: Southeast).

Hourly O_3_ and daily 24 h PM_2.5_ concentrations were obtained from the US Environmental Protection Agency (EPA)’s Air Quality System and Speciation Trend Networks, and Interagency Monitoring of Protected Visual Environments network. We selected sites with at least 10 years of year-round (January–December) data in 1994–2012 and at least 14 daily measurements each month; this yielded 468 O_3_ sites and 62 PM_2.5_ sites (figure 2). We chose 1994 as the starting year due to wider availability of O_3_ measurements. Of note, most PM_2.5_ measurements are available starting 1998. As we ultimately assess health impacts, sites were categorized into seven regions (Industrial Midwest (IM), Northeast (NE), Northwest (NW), Southeast (SE), Southern California (SC), Southwest (SW), Upper Midwest (UM)) as defined by the National Morbidity Mortality Air Pollution Study (NMMAPS) (Samet *et al* 2000). As the national O_3_ standards are based on the annual 4th highest maximum daily 8 h average averaged over 3 years, we computed the daily 8 h max O_3_ metric utilizing a 75% data capture criterion. Specifically, the 8 h max metric was estimated on days with at least 18 of 24 valid 8 h moving averages, which were calculated from at least 6 valid hourly values.

### 2.2. Weather data collection

Daily precipitation frequency (0/1) and hourly temperature (°C), wind speed (m s^−1^), and water vapor pressure (hPa) were obtained from the National Oceanic Atmospheric Administration’s National Climatic Data Center. Daily averages were calculated from at least 18 of 24 hourly measurements. Selected stations had all 19 years of year-round data and at least 21 daily measurements (~75%) per month; this yielded 194 stations measuring surface temperature, wind speed, and water vapor pressure, and 168 stations measuring precipitation frequency (figure S1). Air pollution data were matched to the nearest weather station’s data. Data from 87 weather stations were matched to 468 O_3_ stations with an average distance of 77.3 km, and data from 41 weather stations were matched to 62 PM_2.5_ stations with an average distance of 30.4 km (table S1). Matching distance between PM_2.5_ and weather stations were slightly higher (37.2 km) for precipitation frequency data.

### 2.3. Weather penalty calculation

For each region and season (cold and warm), weather associated changes in O_3_ and PM_2.5_ were quantified by estimating: (1) the magnitude of the unadjusted and weather-adjusted pollution trends using generalized additive models (GAMs), (2) the magnitude of the trend differences (i.e., the weather-related penalty), and (3) the standard error of the trend differences through bootstrap methods (see figure S2 for schematic overview). To estimate national averages, region-specific trends and penalties were meta-analyzed accounting for within- and between-region variability (Berkey *et al* 1998).

#### 2.3.1. Regional air pollution trends

First, regional-level GAMs were applied to estimate the long-term trends of daily PM_2.5_ and O_3_ concentrations, with and without adjusting for weather parameters. Many trend analysis studies have employed GAMs to adjust for inter-annual meteorological variation using smoothing spline functions (Camalier *et al* 2007, Zheng *et al* 2007, Pearce *et al* 2011). We stratified the trend analysis into warm (May–October) and cold (November–April) months, as O_3_ exhibited dichotomous trends (figure S3). For each of the seven regions and season (cold/warm), the unadjusted and weather-adjusted trends were calculated from the following GAMs, using the R statistical package (R Development Core Team 2011):

(1) $${\left(O_{3}\right)}_{ij}=\beta_{0}+\beta_{1,\text{unadjusted}}{\text{ year}}_{ij}+\gamma {\text{ month}}_{ij}\;+\delta {\text{ weekday}}_{ij}+\varepsilon_{ij},$$

(2) $${\left(O_{3}\right)}_{ij}=\beta_{0}+\beta_{1,\text{adjusted}}{\text{ year}}_{ij}+\gamma {\text{ month}}_{ij}\;+\delta {\text{ weekday}}_{ij}+s_{1}(tmp)+s_{2}(ws)+s_{3}(wvp)+\varepsilon_{ij},$$

(3) $${\left({PM}_{2.5}\right)}_{ij}=\beta_{0}+\beta_{1,\text{unadjusted}}{\text{ year}}_{ij}+\gamma {\text{ month}}_{ij}\;+\delta {\text{ weekday}}_{ij}+\varepsilon_{ij},$$

(4) $${\left({PM}_{2.5}\right)}_{ij}=\beta_{0}+\beta_{1,\text{adjusted}}{\text{ year}}_{ij}+\gamma {\text{ month}}_{ij}\;+\delta {\text{ weekday}}_{ij}+s_{1}(tmp)+s_{2}(ws)\;+s_{3}(wvp)+s_{4}(prcp)+\varepsilon_{ij},$$

where, (O_3_)_*ij*_ and (PM_2.5_)_*ij*_ represent daily 8 h max O_3_ or daily PM_2.5_ concentrations, respectively, at site *i* and on date *j* and; β_0_ is the intercept and β_1,unadjusted_ and β_1, adjusted_ estimate the linear unadjusted and weather adjusted pollutant trends (ppb per year or µg m^−3^ per year) in 1994–2012 for a specific region and season. The smoothing spline function, denoted by *s*(), characterizes and adjusts for the nonlinear relationships between weather parameters and daily O_3_ or PM_2.5_ concentrations. The weather-adjusted O_3_ trends adjusted for temperature (tmp), wind speed (ws), and water vapor pressure (wvp), and the weather-adjusted PM_2.5_ trends additionally adjusted for precipitation frequency (prcp). γ and δ are vectors of coefficients that represent monthly and weekday variability, respectively.

#### 2.3.2. Regional weather penalty on air pollution

The adjustment of weather parameters in models (2) and (4) removes the impact of inter-annual weather variation on air pollution trends; in other words, the weather-adjusted trends represent trends that assume weather parameters remained constant during the study period. In comparison, the weather impact is incorporated into the unadjusted trends. Therefore, any differences between the unadjusted and weather adjusted trends are entirely attributable to the impact of long-term weather changes. We obtained the trend differences for each region and season, and refer to them as the weather ‘penalty’:

(5) $$\text{Penalty }(ppb{\text{ year}}^{-1}\text{ or }\mu gm^{-3}{\text{ year}}^{-1})\;=\beta_{1,\text{unadjusted}}-\beta_{1,\text{adjusted}}.$$

A positive penalty (β_1,unadjusted_ > β_1,adjusted_) indicates that an increase in air pollution was associated with long-term weather changes during the study period.

#### 2.3.3. Standard error of regional weather penalty

The standard errors for the trend differences (penalties) were derived by utilizing a block bootstrap procedure (Politis 2003) as the penalties are estimated from two related regression models applied to the same data. Briefly, we created randomized subsets of the actual data (i.e., pseudo-datasets) that accounted for serial correlation structures among O_3_ or PM_2.5_ observations. We utilized a block size of 20 days to create 100 pseudo-datasets for each region and season (1400 pseudo-datasets total for each pollutant). Then, the unadjusted trends, weather-adjusted trends, and penalty were iteratively estimated from each pseudo-dataset. The standard deviations of the distribution of 100 estimates of the unadjusted trend, weather-adjusted trend, and penalty obtained from 100 pseudo-datasets were estimated as the corresponding standard errors.

### 2.4. Weather trends

For each region and season, a general linear regression model was applied to estimate trends of temperature and wind speed, adjusting for monthly variability within a season. Region-specific trends were meta-analyzed to estimate national average trends (Berkey *et al* 1998). Unlike temperature and wind speed, water vapor pressure and precipitation frequency trends were not linear during our study period and exhibited a shift in trends during the latter half of the study period. We estimated trends of water vapor pressure and precipitation frequency during 1994–2003 (10 years) and 2004–2012 (9 years). A binomial regression model was utilized to estimate precipitation frequency trends.

### 2.5. Mortality impact estimation

Mortality calculations were conducted using EPA’s Environmental Benefits Mapping and Analysis Program (BenMAP) ver.4.0.66 (Abt Associates Inc. 2011). For each NMMAPS region, we estimated three different annual mortality counts: mortality averted by observed improvements in air quality (applying unadjusted pollution trends), mortality that would have been averted if weather conditions remained constant (applying weather-adjusted pollution trends), and the excess mortality resulting from the weather penalty. To estimate mortality associated with unadjusted and weather-adjusted pollution trends and the weather penalty, we applied the following health impact function for each region and season:

(6) $$\Delta \text{Mortality}=y_{0}\times (1-e^{-\beta \times \delta })\times Pop,$$

where, β is the mortality risk coefficient, δ is the regional air quality change of interest (unadjusted trend, weather-adjusted trend, or weather penalties), Pop is the exposed population size, and *y*_0_ is the baseline mortality incidence rate. We utilized BenMAP’s library of county-level population and mortality incidence data for 2010. The county-level mortality estimates were aggregated to the regional level. We applied national risk coefficients from epidemiological cohort studies on chronic O_3_-related respiratory mortality (Jerrett *et al* 2009) and PM_2.5_-related chronic mortality from cardiopulmonary disease and lung cancer (Krewski *et al* 2009) in adults (≥30 years). These studies reported a 3.9% increase (95% CI: 1.0%, 6.7%) in mortality risk per 10 ppb increase in O_3_ and a 5.8% increase (95% CI: 3.8%, 7.8%) in risk per 10 µg m^−3^ increase in PM_2.5_. The risk coefficients selected for this analysis are consistent with those used by the US EPA in recent regulatory analyses (US EPA 2006, 2008) as well as other papers (Tagaris *et al* 2009, Anenberg *et al* 2010). National mortality risk estimates were used to estimate regional mortality changes associated with regional air quality changes, due to high uncertainty of regional mortality risk estimates reported by epidemiological studies compared to the pooled national risk estimate.

To be consistent with air pollution metrics utilized in these studies, we re-estimated trends and weather penalties using year-round (January–December) PM_2.5_ and warm season (April–September) 1 h max O_3_ metrics. To obtain the uncertainty around mortality estimates, we accounted for standard errors of both the health risk coefficients and air quality change of interest (unadjusted trend, weather-adjusted trend, or weather penalty) using the multivariate delta method (Agresti 2012):

(7) $$\text{Variance}={\left(\partial f/\partial \beta \right)}^{2}\times \text{Variance}(\beta )\;+{\left(\partial f/\partial \beta \right)}^{2}\times \text{Variance}(\delta ),$$

where, ∂*f*/∂ represents the partial derivative of equation (6) with respect to either β or δ. The variances of β and δ are derived from epidemiological studies and the bootstrap procedure, respectively.

## 3. Results and discussion

### 3.1. Raw monthly average time series of O_3_ and PM_2.5_

**Figure 3:**
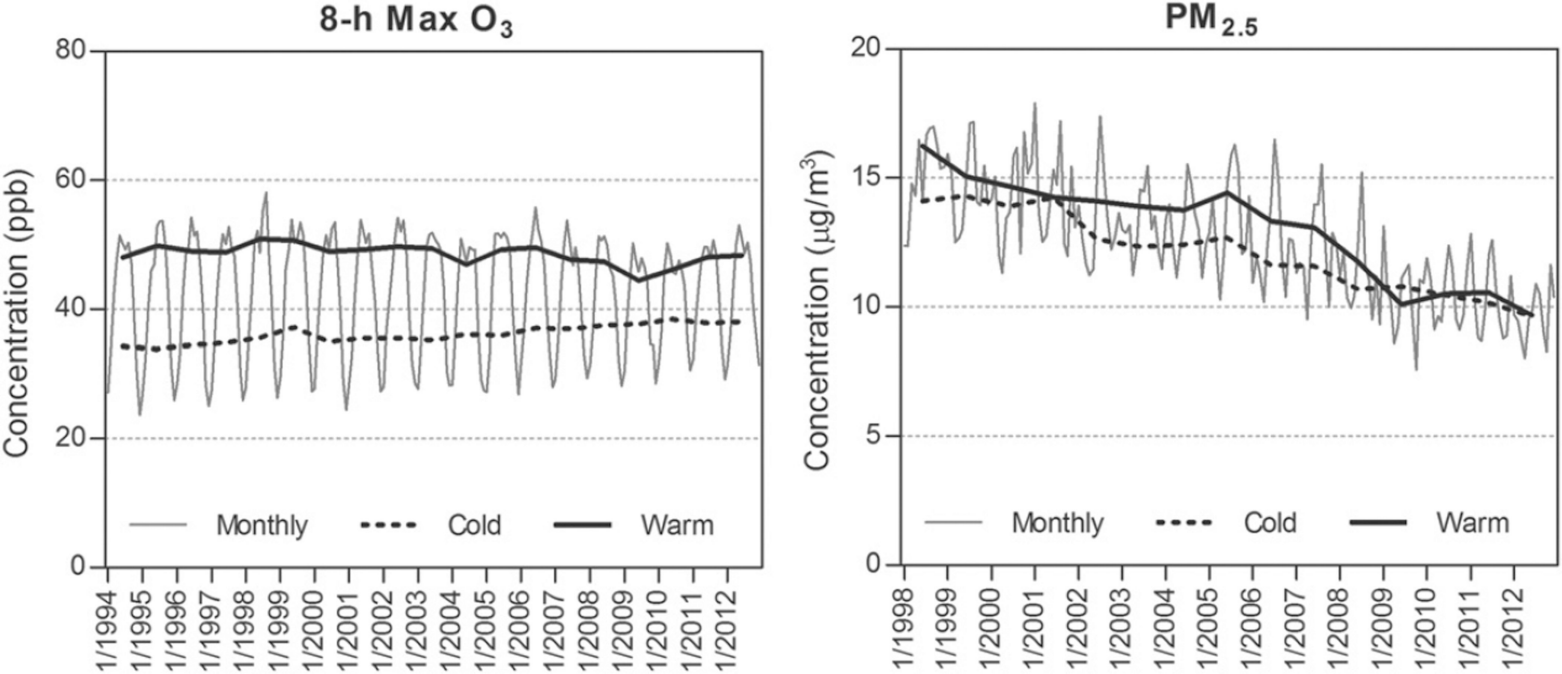
National monthly mean concentration time series of 8 h max O_3_ and PM_2.5_. The cold season (November–April) and warm season (May–October) monthly means were averaged to estimate seasonal time series.

To assess the air quality measurement data from selected monitoring sites (468 O_3_ and 62 PM_2.5_ sites), we estimated the raw national monthly averages of PM_2.5_ and 8 h max O_3_, as well as cold (November–April) and warm (May–October) season averages in 1994–2012. During our study period, significant PM_2.5_ decreases were observed, while there was only a modest change in 8 h max O_3_ (figure 3). Trends derived from regression analyses are discussed in subsequent sections. Most PM_2.5_ measurements were available starting 1998 and showed considerable decreases in both warm and cold months from 16.2 to 9.7 µg m^−3^ and 14.1 to 9.6 µg m^−3^ in 1998–2012, respectively. These drastic PM_2.5_ decreases were consistent with the national PM_2.5_ trends and concentrations reported by the US EPA (US EPA 2014b). In contrast, the cold season average of daily 8 h max O_3_ increased from 34.3 to 38.0 ppb in 1994–2012, while warm season 8 h max O_3_ decreased from 48.0 to 44.4 ppb in 1994–2009 then increased slightly there-after. Recent warm season O_3_ increases are also reported by the US EPA (US EPA 2014a). These time series reflect the raw air pollution concentrations resulting from a combination of emissions and weather conditions. In the following sections, we estimated the proportion of changes in air pollution attributable to changes in weather conditions.

### 3.2. Trends of weather parameters

To investigate the weather-associated changes in air quality, we analyzed data on temperature, water vapor pressure, wind speed, and precipitation frequency from over 200 weather stations (figure S1); prior observational and model perturbation studies identified these four parameters to be among the most important meteorological determinants of O_3_ and PM_2.5_ concentrations (Dawson *et al* 2007a, 2007b, Jacob and Winner 2009, Fiore *et al* 2012). We assessed the raw time series and trends of each weather variable, and summarized the trends in table 1.

#### 3.2.1. Temperature trends

**Figure 4:**
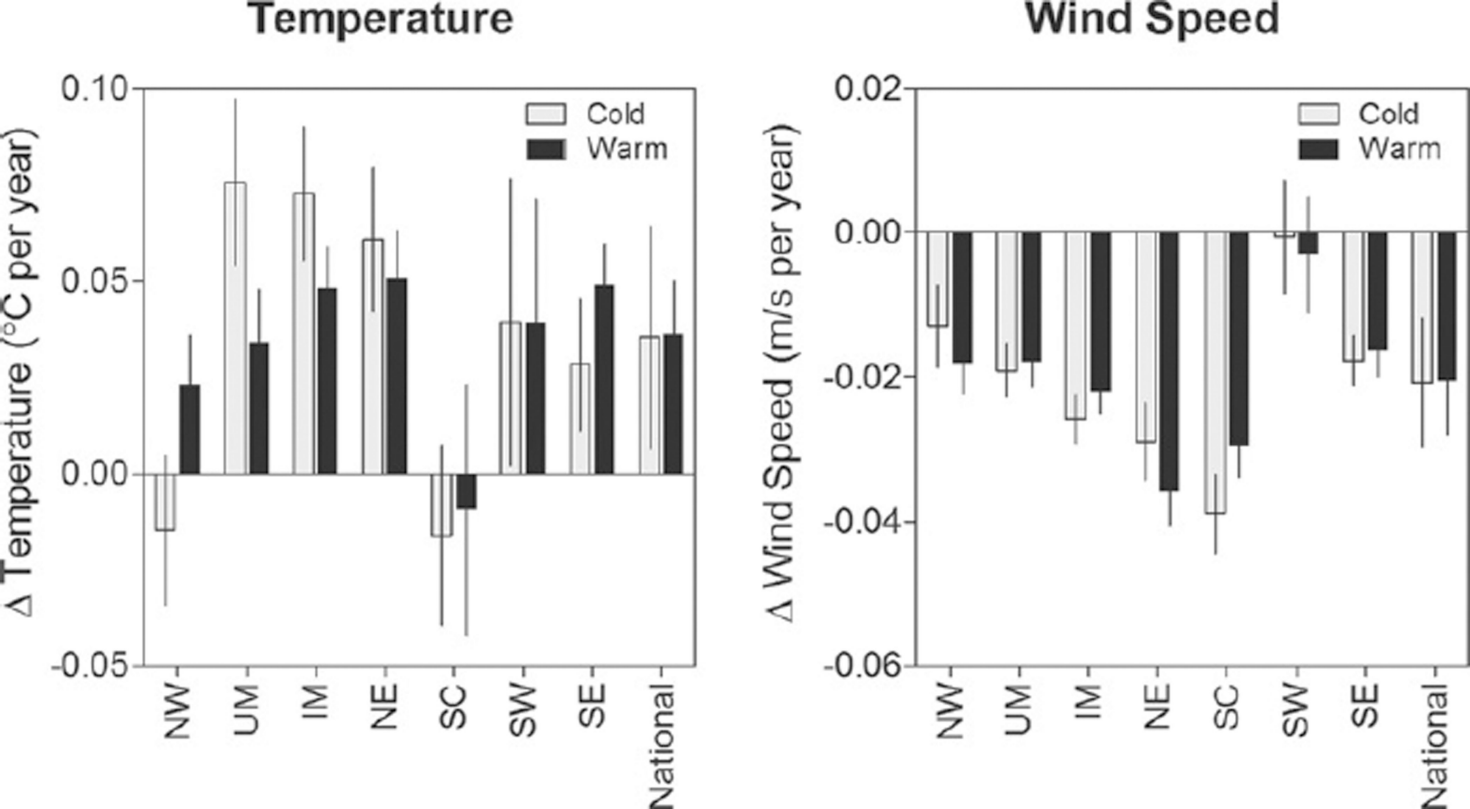
Changes in temperature (°C per year), wind speed (m s^−1^ per year) in 1994–2012 by region and season. The 95% confidence intervals are shown.

During the study period (1994–2012), temperature increases were observed year-round in most regions (figure 4). A national meta-analysis of regional temperature trends yielded a statistically significant increase by 0.035 °C (0.64%) per year in the cold months and 0.036 °C (0.18%) per year in the warm months. Percent changes are relative to national average temperatures. The temperature increases during our study period were in agreement with those reported in the literature (Isaac and van Wijngaarden 2012). The greatest temperature increases were observed during the cold season in the Northern regions (e.g., UM, IM, NE). The West Coast regions (NW and SC) exhibited the least or no temperature change.

#### 3.2.2. Wind speed trends

Decreases in ground level wind speed were observed in all regions except in the SW (figure 4). Meta-analysis of regional wind speed trends yielded a national decrease in wind speed by 0.021m s^−1^ (0.49%) per year in the cold season and 0.021m s^−1^ (0.57%) per year in the warm season. The magnitude of wind speed declines during our study period were in agreement with those reported in the literature (Pryor *et al* 2009). In addition, wind speed is expected to continue to decline, as frequency and duration of stagnation episodes increase in the future climate (Mickley 2004).

#### 3.2.3. Water vapor pressure trends

Trends of water vapor pressure and precipitation frequency were not linear in many regions during our study period. We estimated regional and national water vapor pressure trends during 1994–2003 (10 years) and 2004–2012 (9 years) (table S2). Nationally, cold season water vapor pressure increased by 0.023 hPa (0.32%) per year in 1994 to 2003 and by 0.008 hPa (0.11%) per year in 2004 to 2012. In contrast, warm season water vapor pressure did not exhibit statistically significant changes in 1994–2003 and decreased by 0.047 hPa (0.30%) per year in 2004–2012. These water vapor pressure trends were in general agreement with those reported in the literature (Isaac and van Wijngaarden 2012). During the cold season, water vapor pressure increased in areas where temperature increased (IM, NE, UM, SE) and decreased in areas where temperature decreased (NW, SC). In contrast, warm season water vapor pressure decreased in 2004–2012 in most regions (NW, UM, IM, SE, SW) while temperature increased.

#### 3.2.4. Precipitation frequency trends

As precipitation provides a main sink for PM_2.5_, we accounted for changes in precipitation frequency in subsequent analyses to estimate the weather penalty on PM_2.5_, but not for O_3_ given the weak evidence on its correlation with precipitation (Dawson *et al* 2007a). Specifically, precipitation frequency is a more relevant metric than the intensity or amount of rainfall (Jacob and Winner 2009, Tai *et al* 2012), as particles are efficiently scavenged through wet deposition (Balkanski *et al* 1993).

During the cold season, precipitation frequency increased in 1994–2003 in all regions (by 1.3% per year on average) and decreased in 2004–2012 in most regions except NW and NE (by 0.35% per year on average). During the warm season, precipitation frequency increased in 1994–2003 in all regions except SC (by 2.6% per year on average) and increased in 2004–2012 in most regions except NW, SC, and UM (by 0.44% per year on average).

### 3.3. Weather penalty on air quality

**Figure 5:**
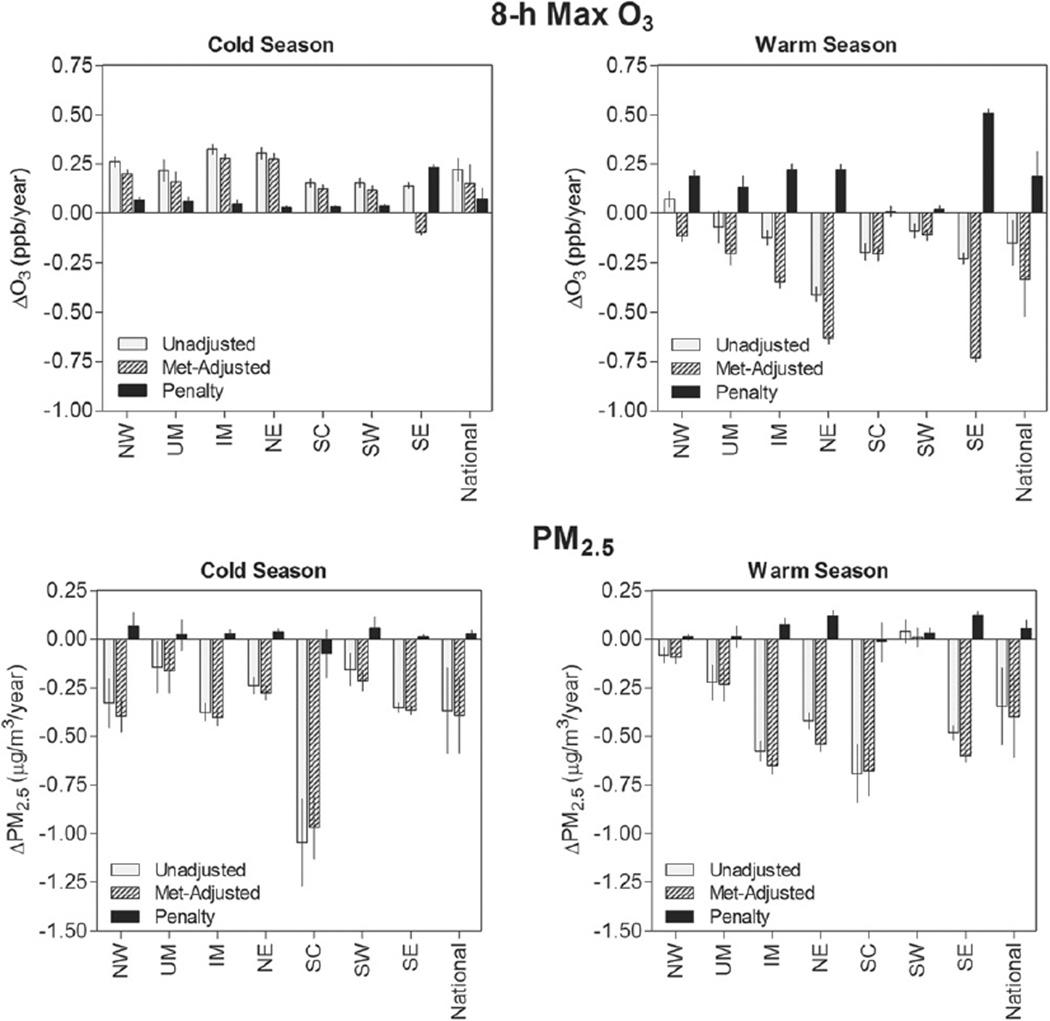
Unadjusted trends, weather-adjusted trends, and weather penalties of 8 h max O_3_ and PM_2.5_ in 1994–2012 by region and season. The 95% confidence intervals are shown.

Weather changes during our study period (1994–2012) were associated with significant increases in daily 8 h max O_3_ and daily PM_2.5_ during both cold and warm seasons, particularly in the Eastern US (NE, SE, and IM) (figure 5). The unadjusted O_3_ and PM_2.5_ trends in each region and season reflect the trends resulting from a combination of weather changes and emission changes. The weather-adjusted trends remove the influence of inter-annual changes in temperature, wind speed, and water vapor pressure (and precipitation frequency as well for PM_2.5_) on air quality. Finally, the differences between unadjusted trends and weather-adjusted trends reflect the impact of long-term weather changes on air pollutant trends (i.e., weather penalty). The weather penalty we estimate includes direct effects of weather conditions (e.g., photochemical reactions), indirect effects (e.g., more heating use on cold days), and effects of other meteorological phenomena with ground-level weather manifestations (e.g., transport of cold, dry air mass).

#### 3.3.1. Ozone trends and weather penalty

Nationally, the cold season daily 8 h max O_3_ increased by 0.22 ppb (or 0.64%) per year, and all regions exhibited cold season O_3_ increases. If temperature, wind speed, and water vapor pressure had not changed during our study period, the cold season O_3_ would have increased by 0.15 ppb per year instead. In other words, weather changes led to additional O_3_ increases by 0.07 ppb per year (95% CI: 0.02, 0.13). During the warm season, O_3_ decreased by 0.15 ppb (0.31%) per year nationally, and decreased in most regions except the NW. If weather conditions had remained constant, warm season-O_3_ would have decreased even more (0.33 ppb per year), reflecting a weather-associated penalty of 0.18 ppb per year (95% CI: 0.06, 0.31). Over 19 years, these amount to a total weather-related increase in daily 8 h max O_3_ of 1.5 ppb in the cold season and 3.4 ppb in the warm season.

Water vapor pressure and temperature were the most important determinants of the absolute O_3_ concentrations and trends in all regions and in both warm and cold seasons. O_3_ is strongly correlated with temperature, as the presence of sunlight increases O_3_ formation. During the warm season, weather penalties were greatest in the Eastern US (SE, NE, and IM), where temperature increases were also greatest. We conducted additional modeling analyses including different permutations of the weather parameters in model (2), which showed that the combination of changes in temperature and water vapor pressure made up the majority of weather penalty on O_3_. Therefore, the nonlinear changes in water vapor pressure during our study period, together with the increases in temperature, resulted in a significant net increase in O_3_ concentrations. Of note, our results were robust to the use of relative humidity instead of water vapor pressure (table S3, figure S4). This combined effect of water vapor pressure and temperature changes was particularly important in the SE, where the highest O_3_ penalties were observed.

Water vapor has competing effects on O_3_ concentrations by facilitating hydroxyl radical production from O_3_ photolysis, which can collectively yield a net O_3_ loss (via photolysis) or net O_3_ production (via hydroxyl radical chemistry). Very dry conditions, however, can cause drought stress and suppress stomatal O_3_ uptake and contribute to the high warm season O_3_ (Vautard *et al* 2005, Solberg *et al* 2008). Therefore, drier and hotter conditions in recent years (2004–2012) may have increased warm season O_3_ concentrations.

Decreases in wind speed were consistently associated with O_3_ increases in the warm season. Low wind speed and high ambient temperature are conditions characteristic of stagnation leading to favorable conditions for O_3_ formation during the summertime (Banta *et al* 1998). In fact, additional analyses showed that the combined effect of wind and temperature were important during the warm season and yielded a statistically significant weather-associated penalty, whereas little or no significant impacts were observed during the cold season.

In summary, much of the weather penalty on O_3_ can be attributed to changes in temperature and water vapor pressure, but the significant decline in wind speeds also contributed to the warm season O_3_ penalty.

#### 3.3.2. PM_2.5_ trends and weather penalty

The weather penalty on PM_2.5_ was relatively smaller than that of O_3_, but statistically significant nonetheless. Nationally, daily PM_2.5_ decreased by 0.37 µg m^−3^ (2.6%) per year during the cold season. Without weather changes, PM_2.5_ would have decreased by 0.39 µg m^−3^ per year, reflecting a weather penalty of 0.03 µg m^−3^ per year (95% CI: 0.01, 0.04). During the warm season, PM_2.5_ decreased by 0.35 µg m^−3^ (2.3%) per year nationally, and would have decreased by 0.40 µg m^−3^ per year without weather changes. This reflects a warm season weather penalty of 0.06 µg m^−3^ (95% CI: 0.02, 0.10) per year. Over a 19-year period, the weather penalty on PM_2.5_ would have been approximately 0.5 µg m^−3^ during the cold season and 1.1 µg m^−3^ during the warm season.

Temperature and wind speed were the most important determinants of PM_2.5_ concentrations and trends in most regions. Most regions (except UM and SC) exhibited statistically significant weather-related PM_2.5_ increases during both seasons. Weather penalties on PM_2.5_ were highest during the warm season in the Eastern US regions (NE, SE, and IM), where warm season temperature increases were also greatest. Temperature increases can have opposing effects on PM_2.5_ by increasing sulfate concentrations through increased oxidation and decreasing nitrate levels due to ammonium nitrate volatilization (Seinfeld and Pandis 2006, Dawson *et al* 2007b). Therefore, the net effect depends by the relative abundance of nitrate and sulfate. Sulfate typically makes up a significant proportion (30–60%) of the PM_2.5_ mass composition in the Eastern US regions due to high sulfate emissions from coal-fired power plants (Hand *et al* 2012). Therefore, this is consistent with temperature-related PM_2.5_ increases in the Eastern US, especially as peaks in sulfate concentrations are more common in the warm season. During the cold season, the weather penalty on PM_2.5_ was lower than the warm season penalty despite greater cold season temperature increases.

In contrast, the association between wind speed and PM_2.5_ was more consistently negative in all regions in both warm and cold seasons. Studies have shown that high wind speed is generally correlated with low pollutant levels due to enhanced advection and deposition (Dawson *et al* 2007b). Therefore, a decline in wind speed can contribute to a more favorable condition for particle formation. Nonetheless, the overall weather penalty on PM_2.5_ was relatively smaller than O_3_, which is likely attributable to nonlinear effects as well as competing effects of weather parameters on different PM_2.5_ components.

### 3.4. Mortality impact of weather penalty

In order to characterize the health consequences of weather-associated increases in O_3_ and PM_2.5_, we applied national risk estimates reported by two epidemiological studies on O_3_ and PM_2.5_ associated mortality (Krewski *et al* 2009, Jerrett *et al* 2009). As we analyzed long-term air quality changes, we chose mortality risk estimates from chronic (rather than acute) air pollution health effects studies. We re-estimated regional air pollution trends and penalties using air pollution exposure metrics consistent with those of the epidemiological studies (figure S5).

**Figure 6:**
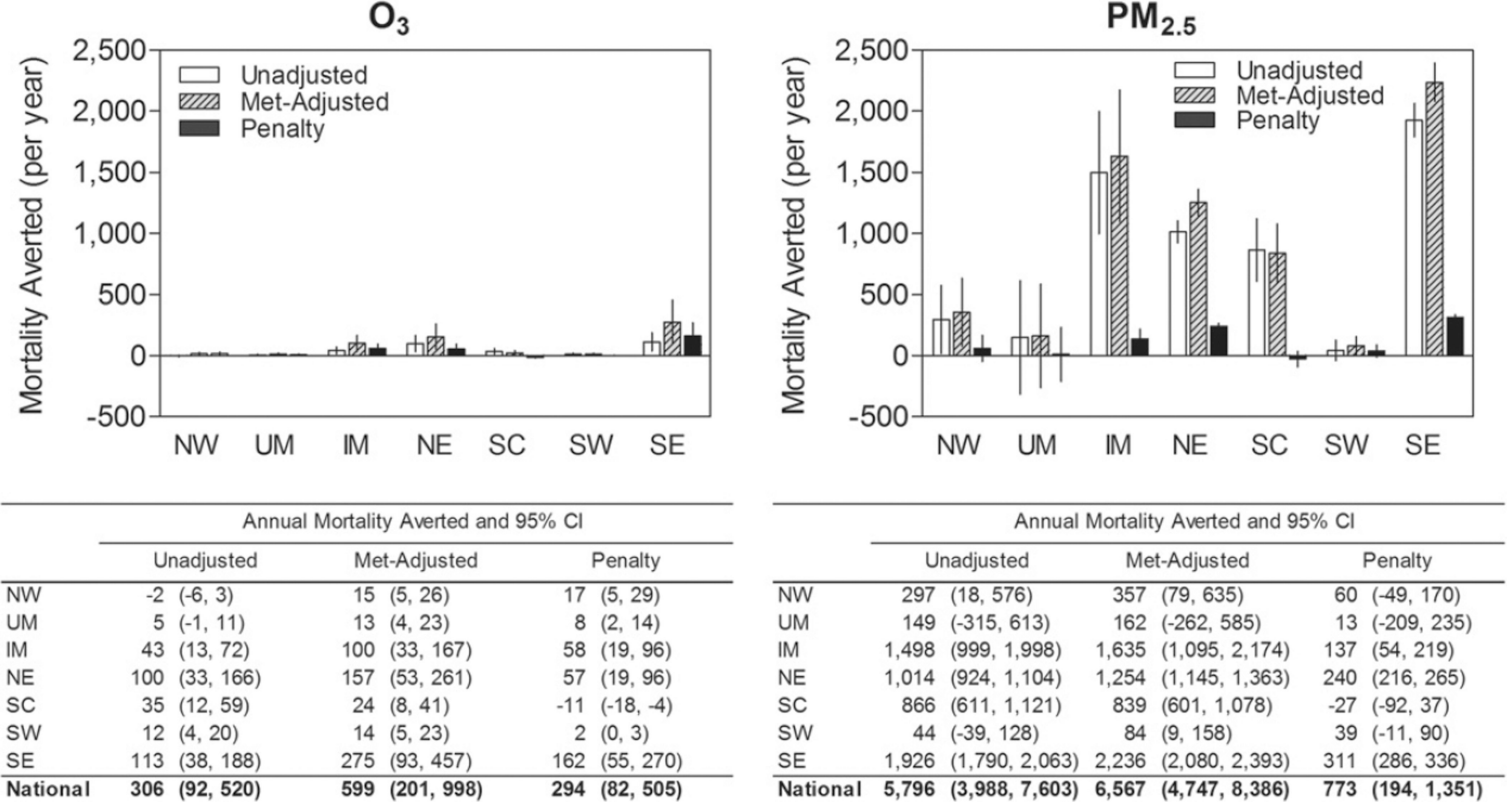
Annual mortalities averted in 1994–2012 as a result of unadjusted and weather-adjusted trends in O_3_ and PM_2.5_, and their difference (penalty) by region. The 95% confidence intervals are shown.

The magnitude of the regional mortality estimates depends on both regional air quality changes and size of population exposed. As such, weather-related penalty on air quality had the greatest mortality impacts in the Eastern US (NE, SE, and IM), where both population size and weather penalties were highest (figure 6). Nationally, 6100 (95% CI: 4100–8100) deaths were averted annually because of air quality improvements during our study period. However, if weather conditions (i.e., temperature, wind speed, water vapor pressure) had not changed, even more deaths, totaling 7200 (95% CI: 4900–9400) annually, would have been avoided. Therefore, weather-related increases in O_3_ and PM_2.5_ were associated with 1100 (95% CI: 300−1900) excess deaths annually. Over a 19-year period, this would amount to approximately 20 300 excess deaths attributable to the weather penalty on air quality.

The weather penalty was associated with 290 annual deaths related to O_3_ and 770 annual deaths related to PM_2.5_. Even though the weather penalty on O_3_ was relatively greater, weather-related PM_2.5_ increases yielded 160% more excess deaths, as PM_2.5_ exposure has a greater mortality effect per unit than O_3_ exposure. While this suggests that weather-associated increases in PM_2.5_-related mortality may continue to be greater, projections for PM_2.5_ and consequently its future health impacts are much more uncertain than those of O_3_.

### 3.5. Limitations

There are several important limitations to our study that affect the magnitude of the weather penalty and mortality estimates. First, in effort to maximize the completeness of weather data to minimize air quality data loss, the weather stations and air pollution monitors were often not co-located. As we aimed to estimate the impact of long-term changes of weather conditions, we assumed that long-term weather trends are similar within the range of distances of our matched sites. A larger distance between air pollution and weather stations is more likely to yield a weaker association, and subsequently an underestimation of the weather penalty.

To further minimize air quality data loss, we also applied a 75% data capture criterion for creating daily O_3_ metrics. While this is the EPA’s minimum data completeness requirement, a non-random pattern of missing hourly values may be a source of bias. In our prior work, we estimated O_3_ trends by hour of the day and season within each region, and found very consistent diurnal and seasonal pattern of trends across all regions (Jhun *et al* 2015). This robust pattern of hourly trends across all seven regions reflect that missing hourly values did not substantially affect the estimation of trends. Furthermore, the effect of missing values on estimating the weather impact on trends (i.e. penalty) is likely even less. Other limitations discussed below are much more likely to be important.

Second, we estimated linear air pollution trends and trend differences. Ideally, we would estimate non-linear trends and trend differences; however, we were limited by computational capacity necessary for estimating non-linear trend differences and their uncertainty via the bootstrap method. As linear trends are more sensitive to outliers, we re-estimated trends and penalties excluding data from the year 2012, as temperature was unusually high that year. We did not observe any statistically significant difference for O_3_ and PM_2.5_ trend differences (i.e., penalty). PM_2.5_ was more sensitive to the exclusion, but any difference was within the 95% confidence interval as we reported above.

Third, due to computational limitations, we estimated trends and trend differences at a regional, rather than site-level scale. Site-to-site heterogeneity is reflected in the confidence interval of the regional trends and trend differences (i.e., weather penalty) we report. On a regional scale, confidence intervals of weather penalties were much smaller than those of trends. This suggests that while site-to-site variation of air pollution trends may be larger, the impacts of weather changes on trends are less variable between sites, and subsequently, between regions. Since our primary aim was to estimate the weather penalty, the regional-level analyses were appropriate and adequate. Another limitation to regional-level analyses, however, is the differences in the number of sites in each region. While there were large site-to-site differences in actual pollution trends, our results show much less heterogeneity in the weather penalty. Nonetheless, the regional weather penalty we estimate (particularly for regions with only a few PM_2.5_ sites) may not be representative of the entire region due to a limited number of selected sites.

Fourth, we only assessed the impact of three or four weather parameters on air pollution trends. We applied a limited set of weather parameters to maximize completeness of the weather data and feasibility of a national analysis. After reviewing the literature, temperature, wind speed, and water vapor pressure (and precipitation frequency for PM_2.5_) were identified as the most important determinants of O_3_ and PM_2.5_ concentrations. As such, the majority of weather-associated penalty on O_3_ and PM_2.5_ are likely accounted for by these variables. However, there are certainly other weather parameters that could have been included such as cloud cover, transport direction/ distance, and atmospheric mixing height. Many of these additional weather parameters may be strongly correlated with the weather variables already included in our models. The incremental value of accounting for other weather parameters may be minimal, given that our models already explain 30–60% of the daily variability in O_3_ and PM_2.5_ concentrations.

Lastly, regional excess mortality associated with weather penalties has important sources of uncertainty. First, we apply a national mortality risk estimate in the health impact function to estimate the mortality impact of regional air quality changes. The regional risk estimates reported by epidemiological studies were highly variable with wide confidence intervals. Second, the O_3_ mortality estimates only account for mortality in April to September of each year, and may be an underestimate to the extent that there are O_3_-related deaths in October through March. Finally, the baseline population data from 2010 were utilized to estimate annual weather-associated excess mortality. Population size changes during our study period, however, represent a very small fraction of the uncertainty of mortality risk estimates and air quality changes.

## 4. Conclusions

In this study, we quantified past weather impacts on air quality and health using long-term observational data. The weather penalty we estimate includes direct effects of weather conditions (e.g., photochemical reactions), indirect effects (e.g., more heating use on cold days), and effects of other meteorological phenomena with ground-level weather manifestations (e.g., transport of cold, dry air mass). Within the recent two decades, historical changes in weather conditions have had significant impacts on air quality and health. Temperature has increased and wind speed has decreased in most US regions. Weather-associated increases in O_3_ were driven primarily by changes in temperature and water vapor pressure, and weather-associated increases in PM_2.5_ were driven by temperature and wind speed. These weather penalties had significant mortality impacts, with approximately 1100 excess deaths per year attributable to the weather penalty on air quality. Excess mortality related to weather-related pollution increases were particularly pronounced in the Eastern US, and were greater for PM_2.5_ even though the weather penalty on O_3_ concentrations was relatively higher. As climate models predict temperature increases, higher frequency of heat waves, and more stagnation episodes, weather-related increases in both O_3_ and PM_2.5_-related mortalities will likely persist in the future. Changes in weather conditions will continue to modify the benefits of emission controls, and this may require additional emissions reductions as more areas exceed air quality standards in the future climate.

## Supplementary Material

Supplemental Materials

## Figures and Tables

**Table 1:** Summary of weather trends.

Weather variable	Season	Trends
Temperature	Cold and warm	↑ (IM, NE, UM, SE, SW)
Wind speed	Cold and warm	↓ (all except SW)
Water vapor press.	Cold Warm	↑ (IM, NE, UM, SE) ↓ (NW, UM, IM, SE, SW)
Precipitation freq.	Cold	↑ (′94-′03),↓ (′04–′12)
	Warm	↑ (all except SC)
