# Supplementary material for: The impact of weather changes on air quality and health in the United States in 1994–2012
Source: Environ Res Lett. Author manuscript; Available in PMC 2016 Aug 24. (PMC4996129; doi:10.1088/1748-9326/10/8/084009)
Supplement: Supplemental Materials [file NIHMS805142-supplement-Supplemental_Materials.pdf]

Supplemental Information

Figure S1. Weather station locations. The number of stations from which temperature (temp), wind speed (ws), and water vapor pressure (wvp) data were obtained is denoted by $n_{temp,ws,wvp}$. The number of stations from which precipitation frequency data were obtained is denoted by n_{rain}. (NW: Northwest, UM: Upper Midwest, IM: Industrial Midwest, NE: Northeast, SC: Southern California, SW: Southwest, SE: Southeast).

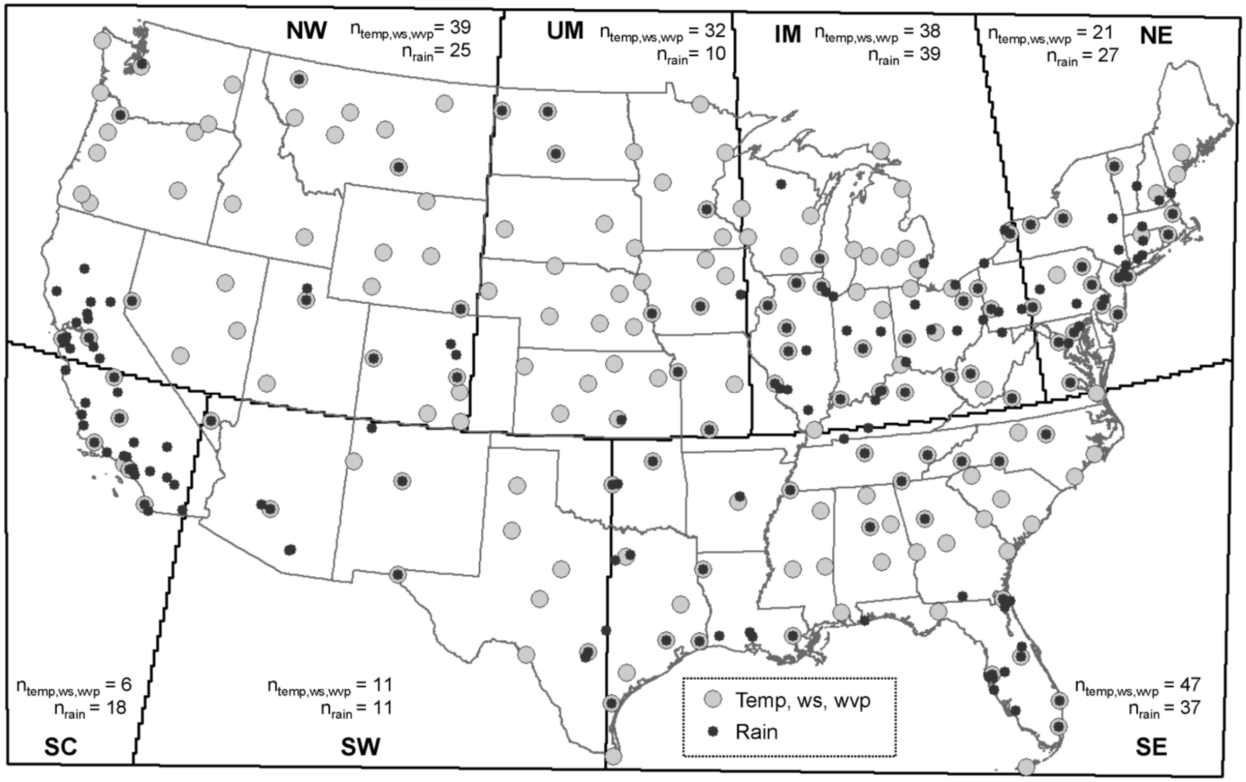

Figure S2. Illustration of weather penalty calculation method for O_3.

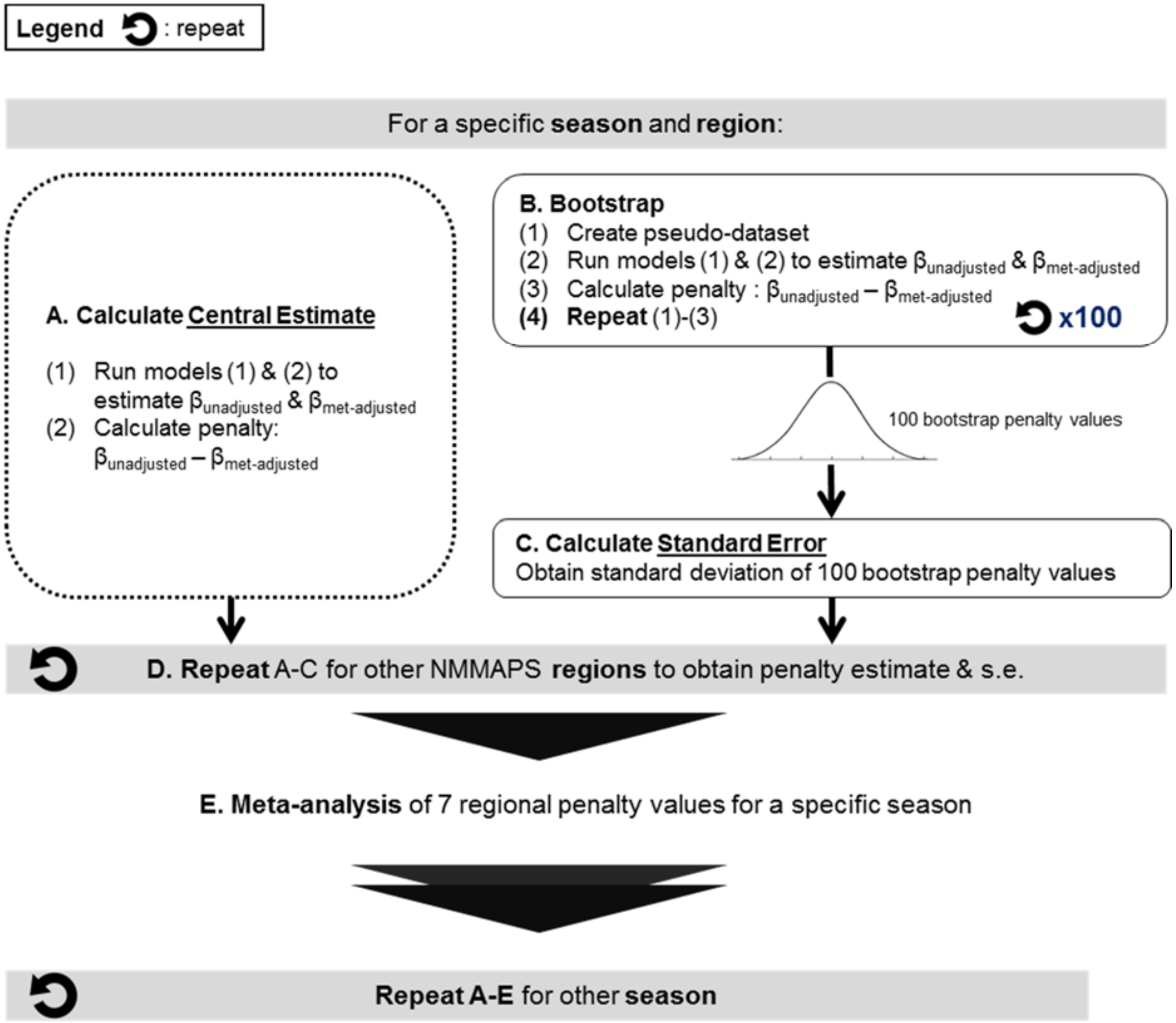

Figure S3. Changes in O_3 and $PM_{2.5}$ by month between 1994-2012.

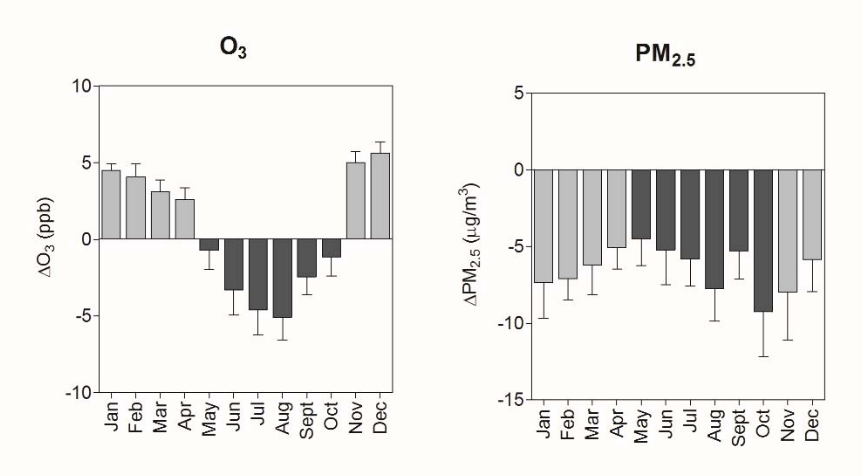

Figure S4. Unadjusted trends, weather-adjusted trends (using relative humidity instead of water vapor pressure), and weather penalties of 8-h max O_3 and $PM_{2.5}$ in 1994-2012 by region and season. The 95% confidence intervals are shown.

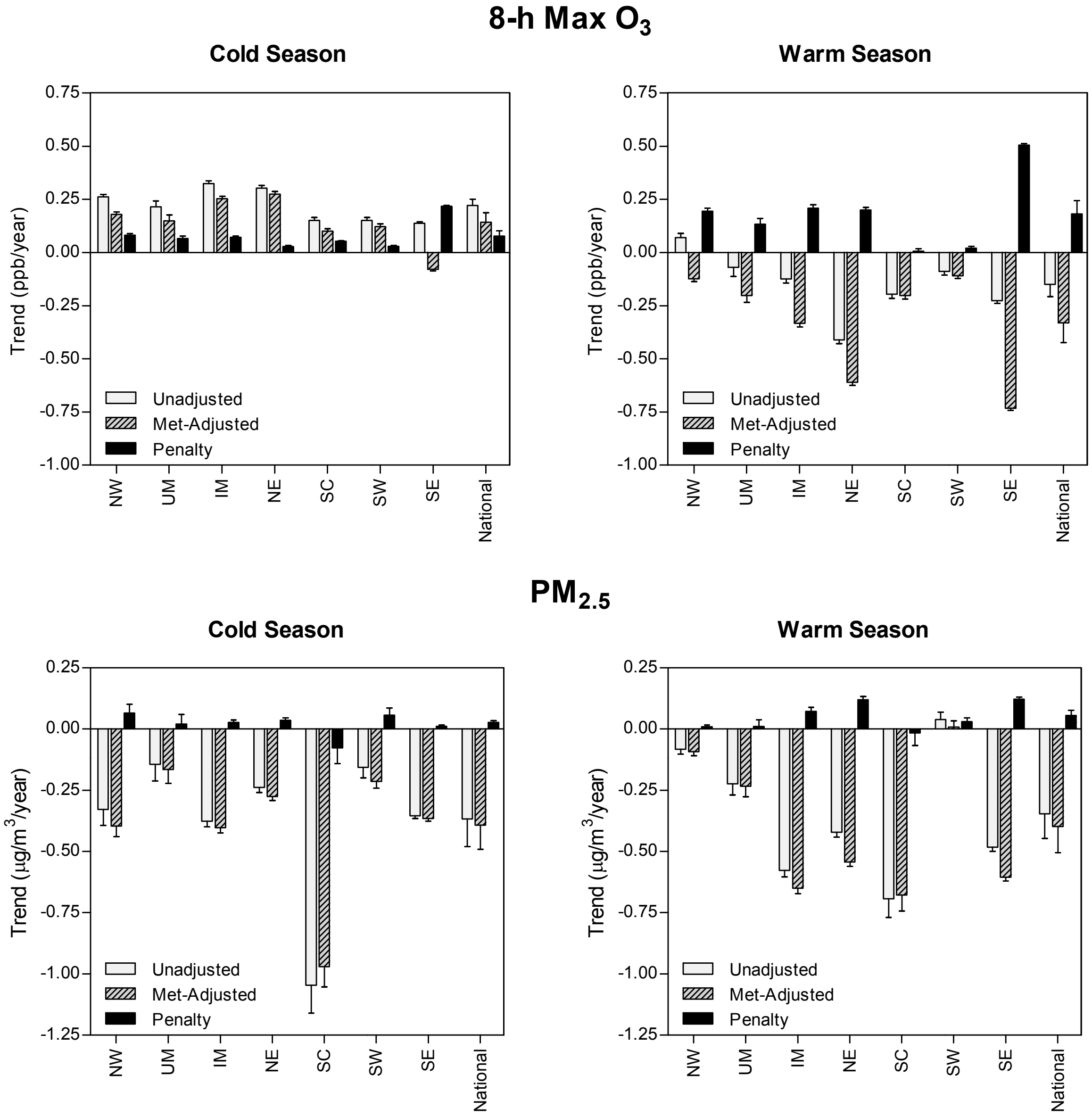

Figure S5. Unadjusted trends, weather-adjusted trends, and penalties of 1-h max O_3 (Apr-Sept) and $PM_{2.5}$ (Jan-Dec) that were applied to estimate mortality averted. The 95% confidence intervals are shown.

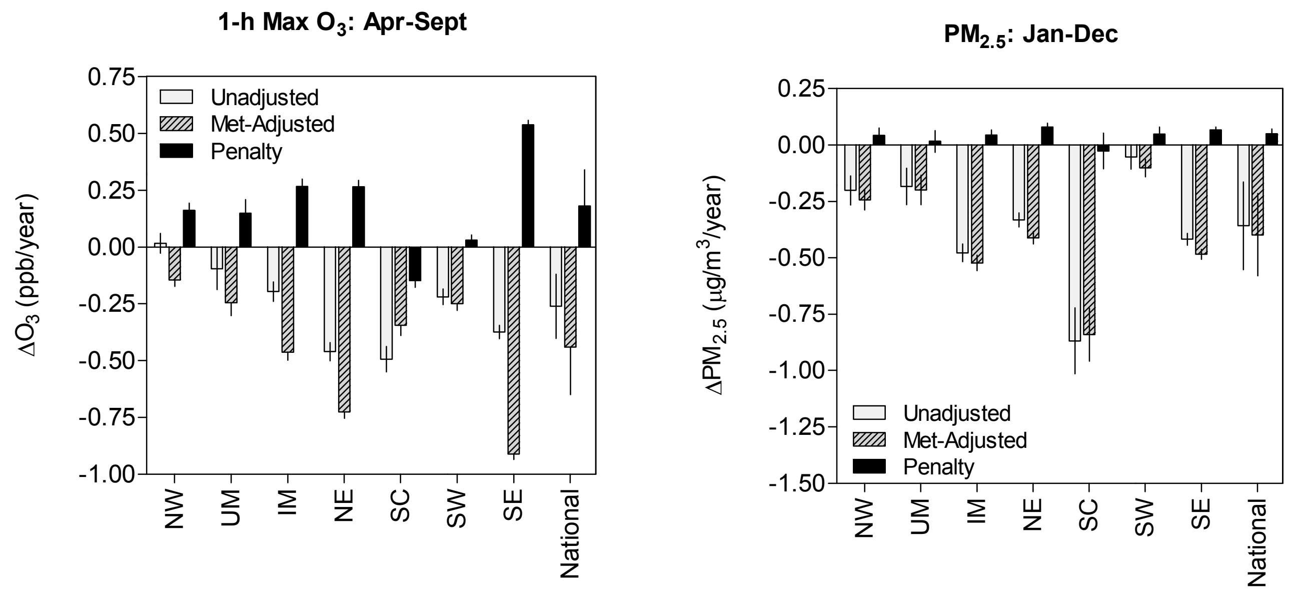

Table S1. Distance between Air Quality Monitoring Stations and nearest Weather Stations (in km)

	Ozone			
**Region**	**N**	**Mean**	**Median**	**(Min, Max)**
Industrial Midwest	30	57.5	38.1	(8.8, 183.9)
Northeast	45	70.0	48.8	(5.1, 194.0)
Northwest	73	117.2	109.5	(2.2, 294.0)
Southeast	144	62.8	43.0	(6.1, 202.5)
Southern California	95	81.5	64.8	(2.9, 225.2)
Southwest	68	71.3	27.8	(5.7, 331.1)
Upper Midwest	13	85.8	68.0	(5.1, 212.3)
**All**	**468**	**77.3**	**53.3**	**(2.2, 331.1)**

	$PM_{2.5}$ (ws, temp, wvp)				$PM_{2.5}$ (prcp frequency)		
**Region**	**N**	**Mean**	**Median**	**(Min, Max)**	**Mean**	**Median**	**(Min, Max)**
Industrial Midwest	11	16.5	16.3	(3.7, 34.3)	14.5	15.5	(3.7, 25.9)
Northeast	14	47.5	37.1	(9.4, 111.7)	36.8	12.5	(4.9, 120.8)
Northwest	5	58.5	53.4	(9.3, 108.5)	30.3	21.6	(5.7, 61.6)
Southeast	24	25.5	17.6	(5.0, 108.3)	58.1	20.3	(6.1, 286.8)
Southern California	4	25.0	12.7	(4.2, 70.2)	11.6	11.7	(4.2, 18.9)
Southwest	3	8.9	10.7	(4.1, 12.0)	8.9	10.7	(4.1, 12.0)
Upper Midwest	1	9.3	9.3	(9.3, 9.3)	9.3	9.3	(9.3, 9.3)
**All**	**62**	**30.4**	**17.4**	**(3.7, 111.7)**	**37.2**	**15.8**	**(3.7, 286.8)**

Table S2. Trends of weather parameters in cold and warm seasons in 1994-2003 and 2004-2012. (*italics*: p-value > 0.05)

		Cold		Warm	
	(per year)	1994-2003	2004-2012	1994-2003	2004-2012
**National**	temp (°C)	0.06	0.06	0.03	0.08
	ws (m/s)	-0.03	-0.02	-0.02	-0.03
	wvp (hPa)	0.02	0.01	*0.00*	-0.05
	prcp (%)	1.29	-0.35	2.61	0.44
**IM**	temp (°C)	0.15	0.13	*0.00*	0.10
	ws (m/s)	-0.02	-0.05	-0.02	-0.02
	wvp (hPa)	0.06	0.03	0.02	-0.02
	prcp (%)	1.08	-1.38	3.23	0.18
**NE**	temp (°C)	0.07	0.20	-0.02	0.11
	ws (m/s)	-0.04	-0.04	-0.04	-0.03
	wvp (hPa)	0.04	0.04	0.03	0.03
	prcp (%)	1.46	0.03	3.21	1.47
**NW**	temp (°C)	-0.02	-0.04	0.08	0.02
	ws (m/s)	-0.02	*0.00*	-0.01	-0.03
	wvp (hPa)	-0.01	-0.04	-0.04	-0.06
	prcp (%)	3.91	0.72	1.88	-0.45
**SE**	temp (°C)	*0.00*	0.06	0.02	0.07
	ws (m/s)	-0.04	-0.03	-0.02	-0.02
	wvp (hPa)	*0.01*	0.05	0.03	-0.05
	prcp (%)	0.19	-0.31	2.23	0.53
**SC**	temp (°C)	-0.09	*-0.02*	-0.06	*-0.02*
	ws (m/s)	-0.05	-0.03	-0.04	-0.03
	wvp (hPa)	-0.08	-0.09	*0.00*	-0.03
	prcp (%)	2.49	-0.83	-1.08	-2.93
**SW**	temp (°C)	*0.00*	0.08	0.05	0.19
	ws (m/s)	*0.01*	*0.01*	0.02	0.01
	wvp (hPa)	0.03	-0.09	0.02	-0.21
	prcp (%)	0.38	-0.83	2.42	0.98
**UM**	temp (°C)	0.19	*0.01*	0.04	0.09
	ws (m/s)	-0.02	-0.03	-0.01	-0.04
	wvp (hPa)	0.05	*0.01*	-0.03	-0.05
	prcp (%)	0.30	-0.44	1.64	-1.04

Table S3. Comparison of national weather-adjusted trends and weather penalties of O_3 and $PM_{2.5}$ using water vapor pressure versus relative humidity.

	Adjusted trends		***Penalty***	
	**wvp**	**rhum**	**wvp**	**rhum**
O_3 cold	0.149	0.142	0.071	0.078
O_3 warm	-0.334	-0.331	0.185	0.181
$PM_{2.5}$ cold	-0.392	-0.392	0.027	0.027
$PM_{2.5}$ warm	-0.397	-0.397	0.056	0.056
